# 

**Published:** 1918-09-14

**Authors:** 


RATES OF SUBSCRIPTION:

Twelve months, 10/10; Six months, 5/5; Three months, 2/9, post free to any address in the United Kingdom. Abroad, Twelve months, 13/-; Six months, 6/6; Three months, 3/3, post free. **THE HOSPITAL "Index" to Volume LXI., October 1917 to March 1918, is now ready, price 3d. per copy, post free.** Address The Manager, THE HOSPITAL, "The Hospital" Building, 28/29 Southampton Street, Strand, London, W.C. 2.

### A Public-Spirited Memorial.

The West Riding County Council proposes that the estate conveyed to them by Mr. and Mrs. G. M. Morrell of Rawdon, as a memorial of their two nephews, should be called "The Mitchell Memorial Home," and be used for discharged sailors and soldiers suffering from tuberculosis. It will accommodate twenty-eight patients. If the need for such a home ceases, it will be used as a school and home for crippled, sick, or tubercular children.

### Matrons' and Sisters' Appointments.

Ashurst Military Hospital, Littlemore, near Oxford.—Miss Mabel Sharp has been appointed assistant matron. She was trained at St. Mary's Infirmary, Highgate Hill, where she was afterwards staff nurse, ward sister, temporary night sister, and home sister. She has also held the post of ward sister at Highfield Military Hospital, Liverpool, night sister at Whipps Cross Infirmary and War Hospital, second assistant matron at the Bristol City Hospital and Sanatorium, and served under the Territorial Force Nursing Service at the 1st Eastern General Hospital, Cambridge.

British Red Cross Hospital, Netley.—Miss Leslie Porter, assistant matron at Poplar Hospital, has been appointed assistant matron, with alternate duty as night superintendent.

Clayton Court Auxiliary Hospital, Liss, Hants.—Miss M. Bowes has been appointed matron. She was trained at St. George's Hospital; has been assistant matron at No. 2 B.R.C.S. Hospital, Rouen, and at the Kingston, Surbiton and District B.R.C.S. Hospital.

Cottage Hospital, Brentford.—Miss Manning has been appointed matron. She was trained at St. George's-in-the-East Parish Infirmary, and was later sister at the Chelsea Infirmary and under the Metropolitan Asylums Board.

Cottage Hospital, Hayes.—Miss Evelyn Key has been appointed matron. She was trained at Walsall and District Hospital, was staff nurse at Dartmouth Cottage Hospital, Westminster Hospital, Shaftesbury, and for two years sister at Exeter and Topham Barracks War Hospital.

Cottage Hospital, Lyme Regis.—Miss Grace E. Owen has been appointed matron. She was trained at Middlesex Hospital, and has since held the post of staff nurse at Woolwich and Plumstead Cottage Hospital, and has done private nursing.

Cumberland and Westmorland Convalescent Institution, Silloth.—Miss Mary Smith has been appointed matron. She was trained at the Leith Public Health Hospital and the Galashiels Hospital, and was later charge nurse at the Thornton Hospital, Fifeshire, and assistant matron at Gilsland Convalescent Home.

Eldwick Sanatorium for Children, Bingley, Yorks.—Miss E. B. Turner has been appointed matron. She was trained at the Infirmary, Isleworth, and was later home sister at the Mount Vernon Hospital for Consumption, Northwood. She is a member of Queen Victoria's Jubilee Institute for Nurses.

Erith Cottage Hospital, Kent.—Miss Emily Alice White has been appointed nurse-matron. She was trained at St. Mary's Hospital, Paddington, and at the British Lying-in Hospital, Endell Street; has been sister-in-charge of the Infirmary, Royal Orphanage, Wolverhampton; theatre staff nurse at Ilford Emergency Hospital; staff nurse at the Cottage Hospital, Horley; and sister at Ashcombe House Red Cross Hospital, Weston-super-Mare.

Hospital for Diseases of the Nervous System, Paralysis and Epilepsy, Belfast.—Miss Phœbe Livingstone has been appointed matron. She was trained at the Royal City of Dublin Hospital and at the National Hospital for the Paralysed and Epileptic, Queen Square; has been assistant matron of the Taunton and Somerset Hospital; has served as sister in Queen Alexandra's Imperial Military Nursing Service Reserve, as massage sister at the Royal Infirmary, Sheffield, and as home sister of the Belfast Nurses' Home. Miss Livingstone is a member of the College of Nursing and an Associate of the Royal Sanitary Institute.

Oakdale Hospital, Blackwood, Mon.—Miss Margaret M. Ellis has been appointed matron. She was trained at the Union Hospital, Cardiff, and has since done military nursing at Birmingham, and district and private nursing at Cardiff.

**NOTE.—Particulars of Appointments for insertion in this Column will be welcomed.**

### The Voluntary Hospitals and the Ministry of Health Bill.

We are asked to state that the pamphlet "The Voluntary Hospitals and the Proposed Ministry of Health Bill," which has been prepared by Mr. J. Courtney Buchanan, secretary of the Metropolitan Hospital, and circulated by the Council of the British Hospitals Association in connection with the Conference called by the Association for Friday, October 18, has been reprinted and can be obtained from the Scientific Press Ltd., 28 and 29 Southampton Street, Strand, London, W.C. 2, price 6d., or by post 7½d.

